# Lethal and Sublethal Effects of Cyromazine on the Biology of *Musca domestica* Based on the Age–Stage, Two-Sex Life Table Theory

**DOI:** 10.3390/toxics12010002

**Published:** 2023-12-19

**Authors:** Hafiz Azhar Ali Khan

**Affiliations:** Institute of Zoology, University of the Punjab, Lahore P.O. Box. 54590, Pakistan; azhar.iags@pu.edu.pk

**Keywords:** biorational insecticide, insect pest management, cyromazine, *Musca domestica*

## Abstract

Cyromazine is a triazine insect growth regulator insecticide that is recommended for control of *Musca domestica* worldwide. Cyromazine is highly effective in causing mortality of *M. domestica*; however, some aspects of its lethal and sublethal effects on the biology of *M. domestica* are still unknown. The present study explored lethal and sublethal effects on several biological traits and population parameters of *M. domestica*. Concentration–response bioassays of cyromazine against third-instar larvae of *M. domestica* exhibited sublethal and lethal effects from concentrations of 0.03 (LC_10_), 0.06 (LC_25_), and 0.14 (LC_50_) μg/g of a larval medium. Exposure of *M. domestica* larvae to these concentrations resulted in reduced fecundity, survival, longevity and oviposition period, and delayed development of immature stages (i.e., egg hatch time and larval and pupal durations) in the upcoming generation of *M. domestica*. The values of population parameters such as intrinsic rate of increase, finite rate of increase, net reproductive rate, age-specific survival rate and fecundity, and age–stage life expectancy and reproductive value, analyzed using the age–stage and two-sex life table theory, were significantly reduced in a concentration-dependent manner in comparison with the control group. In conclusion, the study highlights the significant effects of cyromazine on the biology of *M. domestica* that could help suppress its population in cases of severe infestations.

## 1. Introduction

Cyromazine, N-Cyclopropyl-1,3,5-triazine-2,4,6-triamine, is a triazine insecticide that belongs to the insect growth regulator (IGR) class of insecticides. It is a biorational insecticide with a novel mode of action that interferes with the molting process in insect pests [1]. The IGR class of insecticides is usually highly selective in their toxicity and has low toxicity toward mammals and nontarget organisms [2]. Cyromazine is a widely used insecticide in animal manure for fly control [3]. Cyromazine is recommended for controlling manure-breeding flies in livestock production units by either spraying it over manure heaps or mixing it with manure where it acts as a larvicide [4,5]. In addition, cyromazine remains active after being applied to chicken manure for house fly control (*Musca domestica* Linnaeus) for up to 10–20 weeks [6] due to its stability in hydrolysis and photolysis [7]. In Pakistan, cyromazine has been used for the management of insect pests in field crops and livestock facilities for many years [3,8,9,10,11].

Toxicity of insecticides against insect pests is generally assessed by the extent of mortality of the exposed species and by measuring median lethal concentrations or doses (LD_50s_ or LC_50s_) of insecticides. However, these measurements are not enough to estimate the actual effects of applied insecticides on the biology and population dynamics of insect pests [12]. Insecticides applied to insect pests may affect their biology by impairing neurophysiological and biochemical processes, immunity, development, reproduction, fecundity, survival and longevity, and development of resistance to insecticides, mainly via lethal and sublethal effects of insecticides [13,14]. However, these effects are variable within the same class of insecticides, between different classes, and the insect species in question. For instance, imidacloprid (a neonicotinoid) negatively affected the biological parameters (survival rate, fecundity, development, etc.) of *Nilaparvata lugens* Stål [15] and *Spodoptera litura* (Fabricius) [16]. In contrast, imidacloprid induced hormesis effects in *Aphis gossypii* Glover [17], *Myzus persicae* (Sulzer) [18], and *Metopolophium dirhodum* (Walker) [19]. Hence, it is important to study the lethal and sublethal effects of insecticides on the biology of insect pests instead of mere reliance on mortality data in order to characterize the toxicity of insecticides and devise pest management programs.

*M. domestica* is an economic pest of households and livestock worldwide. This pest transmits pathogens of several diseases in animals, including human beings [20]. Studies revealed that cyromazine has high insecticidal activity against larvae of *M. domestica* [4,11,21,22,23,24]. However, there is a paucity of information on the lethal and sublethal effects of cyromazine on the biology of *M. domestica*. Previously, Jemâa and Boushih [25] reported that there was a significant reduction in fecundity and adult emergence in *Ceratitis capitata* (Wiedemann) following exposure to cyromazine in comparison with the control group.

Assessing the lethal and sublethal effects of insecticides on insects’ biology can be carried out using the age–stage, two-sex life table analysis, which is a comprehensive tool and can be useful for decision making in pest management programs [26]. In the present study, the lethal and sublethal effects of cyromazine on several biological traits and life table parameters of *M. domestica* were systematically investigated. The data of biological traits and population parameters were subsequently analyzed using the age–stage, two-sex life table theory to identify the potentially lethal and sublethal effects of cyromazine.

## 2. Materials and Methods

### 2.1. Toxicity Assessment of Cyromazine against M. domestica

An *M. domestica* strain was acquired from the Department of Entomology, University of Punjab, Lahore (31.5204° N, 74.3587° E), and used for toxicological evaluations. The strain was maintained under laboratory conditions for the last 11 years, without exposure to pesticides, on a sugar-milk-based diet following a well-established protocol [27,28]. Technical-grade cyromazine (purity 99.1%; Chem Service Inc., West Chester, PA, USA) was used to assess its toxicity against *M. domestica*. The toxicity of cyromazine was assessed following the methodology described in our previous reports [3,29]. Briefly, the toxicity of cyromazine against *M. domestica* was determined by mixing it into the larval diet. The ingredients of the larval diet consisted of sugar, powdered milk, yeast, grass meal, and wheat bran in the ratio of 3:3:10:20:40 by weight, respectively, which were mixed by adding water ad libitum. Cyromazine was dissolved in 5% aqueous acetone to make different concentrations that resulted in >0% and <100% mortality. A 2.5 mL of cryromazine–acetone solution was added into a 25 g larval diet to provide final concentrations of 0.03, 0.06, 0.12, 0.25, 0.50, 1.00, and 2.00 µg of cyromazine per gram of the larval diet. These concentrations were used to calculate the median lethal (LC_50_) and sublethal concentrations (LC_10_ and LC_25_) of cyromazine. In the control group, the larval diet was treated with aqueous acetone alone. Twenty 3rd-instar larvae of *M. domestica* were added onto the surface of the treated larval diet of a specific concentration or control per replicate. All the bioassays were repeated three times under the conditions of 26 ± 2 °C temperature, 65 ± 5% relative humidity, and 12: 12 h light/dark photoperiod. The mortality of the introduced larvae was observed 96 h post treatment and the larvae unable to develop into pupae and/or that failed to respond when touched with a camel hair brush were considered dead.

### 2.2. The Effects of Lethal and Sublethal Concentrations of Cyromazine on the Biology of M. domestica

The lethal and sublethal effects of cyromazine on several biological parameters such as fecundity, egg hatching, development of immature stages, adult eclosion, and longevity of different stages were studied following well-established methodologies [30,31] with a few necessary modifications. Three concentrations of cyromazine viz., LC_50_ = 0.14 μg/g, LC_25_ = 0.06 μg/g, and LC_10_ = 0.03 μg/g, were selected from the results of the toxicity assessment experiment (see Section 3) in order to assess their impact on the biology of *M. domestica*. A larval diet treated with LC_50_, LC_25_, LC_10,_ or a control (without cyromazine) was prepared as stated in the above section. A transparent glass beaker (250 mL) was used to introduce sixty newly emerged third-instar larvae into it, which contained a specific concentration of larval diet or control. The pupae that had just formed were removed from the treatment jars and placed in Petri dishes. Each treatment was repeated six times. Newly emerged adults (<24 h old) from each treatment were kept in pairs (10 pairs) and maintained in meshed wooden cages (30 × 30 × 40 cm). In order to conduct a life table study, seventy eggs that had recently been laid by the pairs were picked up from each concentration or control treatment and placed on the larval diet without cyromazine. Developmental time (egg to adult emergence), survival rate, longevity of different stages, and fecundity were observed on a daily basis until the death of individuals. In order to study fecundity, all females developed from the hatched eggs were paired with males from the respective treatment (one pair in one small cage) to see their fecundity until mortality. All the experiments were performed under laboratory conditions of 12:12 h (light: dark) photoperiod, 65 ± 5% relative humidity, and 26 ± 2 °C [31].

### 2.3. Statistical Analyses

Mortality counts of exposed larvae were used to estimate different concentrations (LC_10_, LC_25_, and LC_50_) of cyromazine following the Probit method [32], using PoloPlus 2.0. The population parameters (Table 1) and data on the biological traits of *M. domestica* were analyzed using the TWOSEX-MSChart program [26,33]. The significance of mean values of population parameters and biological traits of *M. domestica* in different treatments were analyzed via paired bootstrap tests using the TWOSEX-MSChart program with 100,000 resamplings [34,35].

## 3. Results

### 3.1. The Toxicity of Cyromazine against M. domestica Larvae

Analysis of bioassays using cyromazine against third-instar larvae of *M. domestica* fitted the Probit linear model (Table 2). Toxicity values of cyromazine that resulted in 10 (LC_10_), 25 (LC_25_), and 50% (LC_50_) mortality of exposed larvae were estimated as 0.03, 0.06, and 0.14 μg/g of larval medium, respectively.

### 3.2. The Effects of Lethal and Sublethal Concentrations of Cyromazine on the Biology of M. domestica

Concentrations of cyromazine had a significant effect on the biology of *M. domestica* (Table 3). A lengthened development period was observed in arenas treated with different concentrations of cyromazine than in the control group. For instance, the egg hatch period was significantly longer (1.76 d) where *M. domestica* was exposed to LC_50_ followed by LC_25_ (1.48 d) and LC_10_ (1.31 d) in comparison with the control group (1.12 d) (F = 23.9; df = 3, 249; *p* < 0.01). Larvae of *M. domestica* took 7.80 d to convert into pupae in LC_50_ treatment followed by 6.80 and 5.96 d in LC_25_ and LC_10_ treatments, respectively, while the larvae took 5.40 d in the control group (F = 72.7; df = 3, 190; *p* < 0.01). The duration of the pupal stage was 7.43 d in the LC_50_ treatment followed by 6.22, 5.20, and 4.33 d in the LC_25_, LC_10,_ and control treatments, respectively (F = 125; df = 3, 190; *p* < 0.01). In short, *M. domestica* treated with the LC_50_ level of cyromazine took 17.00 d to become adult followed by 14.49 and 12.44 d in the LC_25_ and LC_10_ treatments, respectively, while the shortest preadult duration (10.82 d) was observed in the control treatment (F = 196; df = 3, 190; *p* < 0.01). The longevity of all individuals (36.31 d) (F = 32.1; df = 3, 206; *p* < 0.01), female alone (41.79 d) (F = 26.7; df = 3, 97; *p* < 0.01), and male alone (41.30 d) (F = 5.41; df = 3, 89; *p* < 0.01) was the highest in the control treatment, while *M. domestica* treated with the LC_50_ treatment had the lowest longevity followed by the LC_25_ and LC_10_ treatments. The preoviposition period in females of *M. domestica* increased with the increase in the concentration of cyromazine (F = 11.8; df = 3, 97; *p* < 0.01); however, the reverse was observed for oviposition days with no significant difference among the LC_25_ and LC_10_ and control treatments (F = 7.2; df = 3, 97; *p* < 0.01). Cyromazine concentrations also negatively affected fecundity (F = 3.21; df = 3, 97; *p* < 0.05) and preadult survival (F = 5.2; df = 3, 206; *p* < 0.05) in comparison with the control group. *M. domestica* females treated with LC_50_ of cyromazine laid 332.31 eggs/female followed by 390.71, 414.00, and 439.70 eggs/female in the LC_25_, LC_10_, and control treatments, respectively (Table 3).

The analysis of population parameters such as finite rate of increase (λ) d^−1^, intrinsic rate of increase (r) d^−1^, net reproductive rate (*R_0_*) of offspring, and mean generation time (*T*) d using the bootstrap technique also exhibited a significant effect of cyromazine treatments compared with the control treatment (Table 4). The values of *λ*, *r*, and *R_0_* significantly reduced with the increase in LC level of cyromazine (*p* < 0.05). Flies treated with different LC levels of cyromazine required a longer generation time than those in the control treatment (*p* < 0.05) (Table 4).

### 3.3. The Effects of Cyromazine on Population Parameters of M. domestica

**Figure 1:**
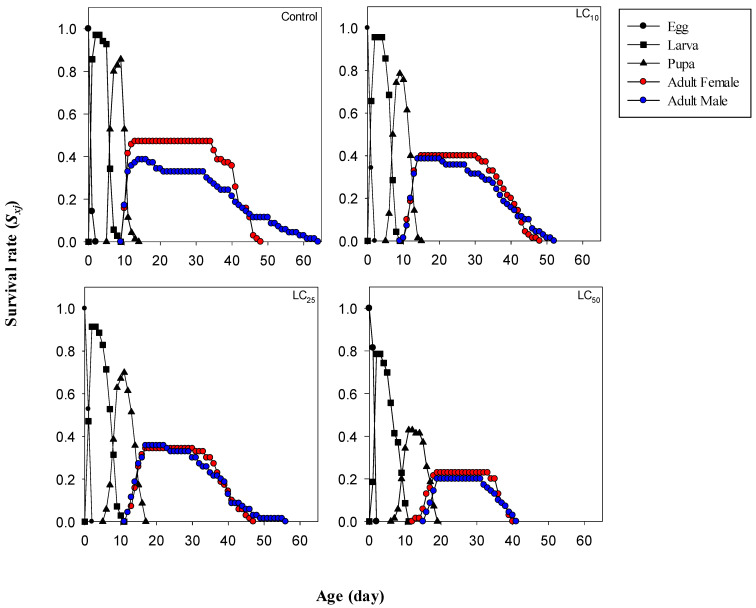
The effects of lethal and sublethal concentrations of cyromazine on age–stage-specific survival rate of *Musca domestica*.

The effects of different concentrations of cyromazine on the age–stage-specific survival rate (*S_xj_*) of *M. domestica* are presented in Figure 1, and the probability of survival of different stages at a specific age is shown by different curves. The analysis revealed that adult female and male *M. domestica* eclosed earlier in the control and LC_10_ treatments (i.e., on the 10th day in both cases) followed by on the 12th day in the LC_25_ treatment. Adult females and males eclosed on the 13th and 16th days, respectively, in the LC_50_ treatment. In addition, the mortality in larval and pupal stages was higher than in other developmental stages in the cyromazine-treated arenas than in the control group.

**Figure 2:**
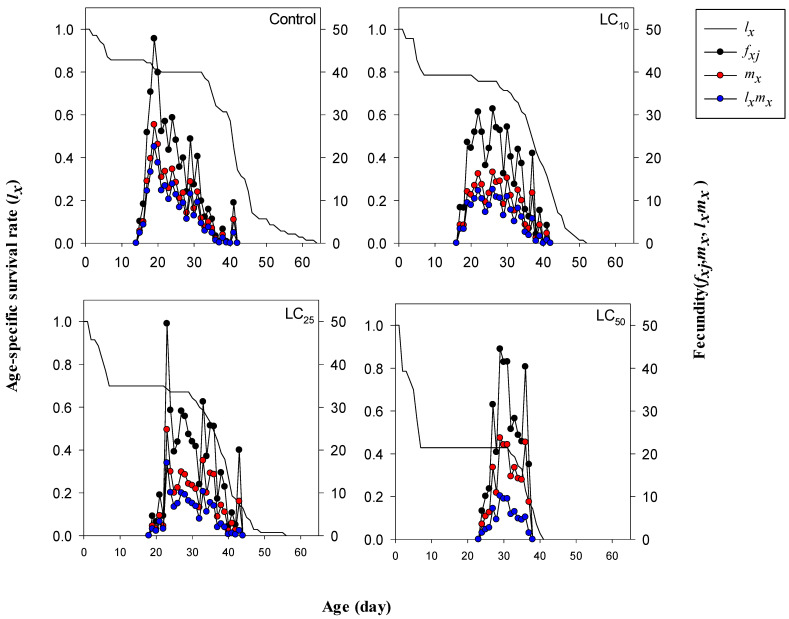
The effects of lethal and sublethal concentrations of cyromazine on age-specific survival rate, fecundity, and maternity of *Musca domestica*.

Exposure of cyromazine to *M. domestica* significantly affected the rate of age-specific survival (*l_x_*), fecundities (*f_xj_*), and maternity (*l_x_m_x_*) (Figure 2). For instance, adult females started to lay eggs on the 15th day in the control group followed by the 17th day in the LC_10_ treatment group, the 19th day in the LC_25_ treatment group, and on 25th day in the LC_50_ treatment group. The overall maternity (*l_x_m_x_*) of *M. domestica* peaked on the 19th, 26th, 23rd, and 29th days in the control, LC_10_, LC_25_, and LC_50_ treatments, respectively, with 22.54, 12.55, 16.97, and 10.16 eggs, respectively.

**Figure 3:**
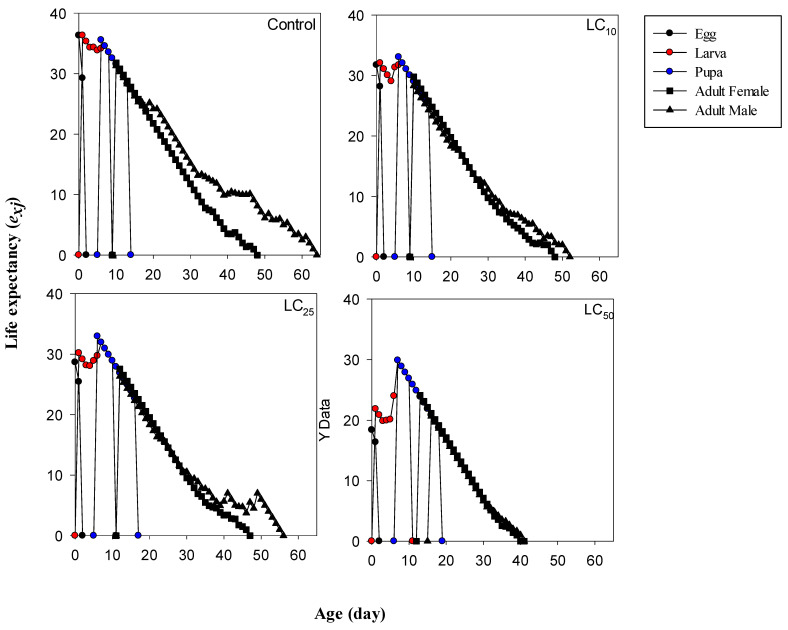
The effects of lethal and sublethal concentrations of cyromazine on the life expectancy of different stages of *Musca domestica*.

The *e_xj_* (life expectancy) values of different life stages of *M. domestica* were also affected by the treatment of cyromazine when compared with that of the control group (Figure 3). For instance, *e_xj_* values of eggs laid by females in the control, LC_10_, LC_25_, and LC_50_ groups were 36.31, 31.73, 28.66, and 18.39 days, respectively, which are completely consistent with those of the longevity values of all individuals (Table 3).

**Figure 4:**
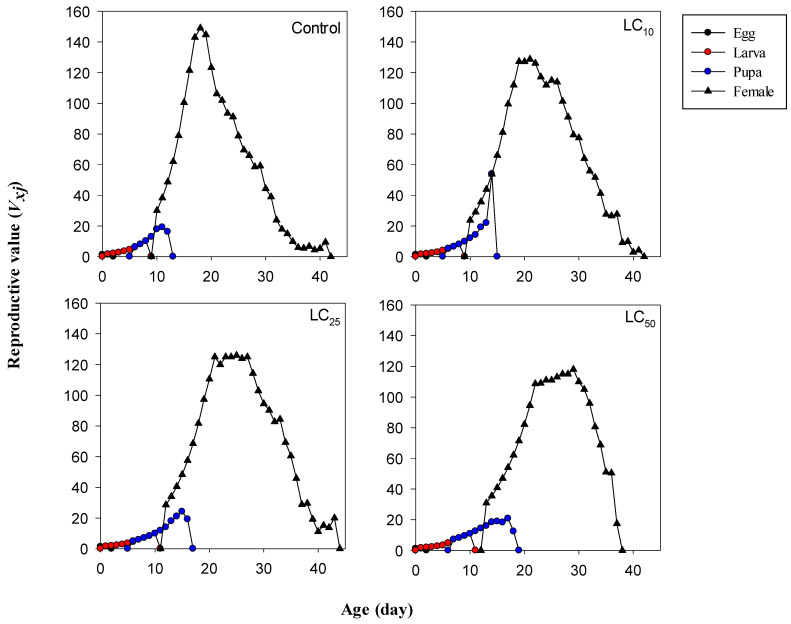
The effects of lethal and sublethal concentrations of cyromazine on the reproductive value of *Musca domestica*.

Similarly, reproductive values “*v_xj_*”, which are used to predict the rate of population growth, were also affected when *M. domestica* was treated with different levels of cyromazine compared with the control group (Figure 4). The *v_xj_* values of *M. domestica* (at age “0”) in the control, LC_10_, LC_25_, and LC_50_ groups were 1.27. 1.23, 1.19, and 1.15 d^−1^, respectively, which is the same as those of the “*λ*” values (Table 4). The curves of *v_xj_* exhibited peaks at 149.03 d^−1^ on the 18th day for the control treatment, 128.75 d^−1^ on the 21st day for the LC_10_ treatment, 125.95 d^−1^ on the 25th day for the LC_25_ treatment, and 120.00 d^−1^ on the 29th day for the LC_50_ treatment.

## 4. Discussion

Biorational insecticides such as cyromazine are usually assumed to be important candidates for managing insect pests of field crops and have medical and veterinary importance [22,41,42]. The market share of biorational insecticides has recently increased worldwide, largely due to the perception that these are safe for the environment and public health compared with conventional insecticides [43]. However, there are a number of environmental concerns linked with biorational insecticides such as the development of resistance in insect pests and nontarget toxicity to predators and pollinators, which need to be considered while planning pest management strategies [44]. Control efficacy of cyromazine has been reported against a number of insect pests such as *Spodoptera littoralis* (Boisd.) [45,46], *Stomoxys calcitrans* (L.) [4,47], *M. domestica* [4,5,11,21,23], *Culex quinquefasciatus* Say [48,49], *Aedes albopictus* Skuse [50,51], *Drosophila melanogaster* Meigen [52], *Liriomyza huidobrensis* (Blanchard) [53], and *Fannia canicularis* (L.) [4].

Besides control efficacy, a few studies have reported cyromazine toxicity on the biological traits of insects. For instance, Fontes et al. [54] reported that the performance of the biological parameters of *Trichogramma achaeae* (Nagaraja and Nagarkatti) was negatively affected after exposure to cyromazine. However, there was a lack of information regarding the toxic effects of cyromazine on the biology of *M. domestica*. Therefore, to explore the ecological influence of cyromazine on *M. domestica*, a systematic approach was followed to determine the lethal and sublethal effects of cyromazine on several biological parameters of *M. domestica*. The results of the present study revealed excellent lethal toxicity toward larvae of *M. domestica*. In addition, toxic effects of cyromazine were also observed in the new generation that was developed from the parent generation whose larvae were exposed to lethal and sublethal concentrations of cyromazine. It is speculated that the exposed larvae were killed by ingesting or coming into contact with the larval medium treated with cyromazine. This effect has also been found when larvae of *M. domestica* were exposed to triflumuron, pyriproxyfen, methoprene, novaluron, diflubenzuron, and cyromazine [4,55]. Cyromazine exhibited high toxicity to third-instar larvae of *M. domestica*, which is consistent with our previous study [3]. The LC_50_ value of cyromazine against third-instar larvae of *M. domestica* was 0.14 µg/g (Table 2), which was quite lower than pyriproxyfen (0.47 µg/g), diflubenzuron (0.68 µg/g), and methoxyfenozide (0.51 µg/g) [3].

Insect pests under field conditions are usually exposed to insecticides either directly, mainly during insecticidal applications, and/or indirectly, such as sublethal exposures due to residues of insecticides after insecticidal applications [31]. Sublethal exposures either result in insect pest suppression or make them resistant, which often results in their resurgence [56]. Hence, the study of biological responses of insect pests after exposure to lethal and sublethal levels of insecticides is important for the success of pest management programs. The data of the present work clearly demonstrate that lethal and sublethal exposure to cyromazine exerts negative effects on the biology of *M. domestica*, which may be translated into the potential to suppress numbers of *M. domestica* with applications of cyromazine.

Exposure to lethal and sublethal levels of cyromazine resulted in prolonged duration of larval and pupal stages. The mean larval duration in the LC_50_ treatment was 7.80 d in comparison with the control group, where larvae took 5.40 d to convert into pupae. Similarly, pupae took more time to become adults after exposure to cyromazine than in the control group. The prolonged duration of larval and pupal stages of insects after exposure to insecticides might be due to feeding cessation and starvation stress at larval stages, or the imbalance between development and detoxification activities after exposure to insecticides [57,58]. Hence, the development of *M. domestica* was slow in cyromazine treatments compared with the control group.

Exposure of *M. domestica* to cyromazine also resulted in reduced fecundity, lengthened preadult and preoviposition periods, and reduced longevity in a concentration-dependent manner. Such types of negative effects on biology after exposure to insecticides have also been reported in different insect pests [59,60,61]. For example, exposure of *M. domestica* to lethal and sublethal levels of cantharidin and pyriproxyfen exerted negative effects on the performance of biological traits [29,62]. In the present study, the negative effects of cyromazine on the performance of biological traits of *M. domestica* are in line with the concept that the biology of survivors after exposure to insecticide may be changed [63]. The changes might be positive, such as the hormesis phenomenon after exposure to pesticides [64], or negative as has been observed in the present study. An increase or decrease in the fecundity of insect pests following exposure to insecticides may result in the expansion or suppression of insect pest populations, respectively, and have important implications for pest management programs [65,66]. In the present work, reduced fecundity and delayed development of *M. domestica* following exposure to cyromazine are in broad agreement with *C. capitata,* which also exhibited the same following exposure to cyromazine [67].

Cyromazine has been recommended to control *M. domestica*; however, cases of resistance development have also been reported worldwide, including in Pakistan [3,68,69,70]. An *M. domestica* strain (CYR-SEL) was selected with cyromazine under laboratory conditions [3]. The strain rapidly developed 211-fold resistance to cyromazine in comparison with a laboratory-susceptible strain after seven generations in selection experiments. However, resistance to cyromazine was unstable since it declined rapidly when the CYR-SEL strain reared for the next seven generations without exposure to cyromazine. The unstable nature of cyromazine resistance in *M. domestica* suggested that there might be a phenomenon of fitness cost that resulted in the rapid decline in resistance [3]. The weak performance of biological parameters in the present study further strengthened the hypothesis that *M. domestica* exhibits fitness costs following exposure to cyromazine.

Recent advances in ecotoxicology are influencing the evaluation of insecticidal effects in exposed populations. Forecasting the impacts of insecticides on insect populations can be carried out effectively using the age–stage, two-sex life table theory [37,71]. By applying this theory, the potential growth of an insect population can be better assessed by studying the finite rate of increase (*λ*) as well as the intrinsic rate of increase (*r*); both are calculated using data on the biological traits of the insect species in question [72]. These calculations show the impact of the rate of development, survival, and fecundity on the fitness of the population in specific conditions. All of the population parameters of *M. domestica* were affected following exposure to different concentrations of cyromazine, which leads to lower values of *λ*, *r*, and *R*_0_ and a higher value of mean generation time than those in the control group. These results are in broad agreement with our recent report on the lethal and sublethal effects of pyriproxyfen on *M. domestica* [29], where the performance of biological parameters of *M. domestica* was compromised following exposure to pyriproxyfen.

In conclusion, the data of the present study revealed that cyromazine is highly toxic to *M. domestica*. The exposure of *M. domestica* to lethal and sublethal concentrations of cyromazine exerted negative effects on the biology of progeny generation. Lengthened developmental time, reduced fecundity and longevity, and low values of population parameters (*λ*, *r*, and *R*_0_) of *M. domestica* were observed after exposure to different concentrations of cyromazine. Further experiments are required under field conditions to understand the putative negative effects of cyromazine on the biology of natural populations of *M. domestica*.

## Figures and Tables

**Table 1:** Population parameters of *Musca domestica* selected to study the effects of cyromazine *.

Parameter	Equation	Explanation
Intrinsic rate of increase (*r*)	$\sum_{x=0}^{\infty }e^{-r(x+1)}l_{x}m_{x}=1$	“It is the population growth rate as time approaches infinity and population reaches the stable age-stage distribution. The population size will increase at the rate of *e^r^* per time unit. The Euler-Lotka equation was used to calculate the intrinsic rate of increase with the age indexed from 0” [36].
The finite rate of increase (*λ*)	$\sum_{n=1}^{\infty }\left(\lambda^{-\left(x+1\right)}\sum_{j=1}^{m}f_{xj}S_{xj}\right)=1$	“The finite rate of increase (*λ*) is the population growth rate as time approaches infinity and population reaches the stable age-stage distribution. The population size will increase at the rate of *λ* per time unit” [26].
Net reproductive rate (*R*_0_)	$\sum_{x=0}^{\infty }l_{x}m_{x}=R_{0}$	“the total offspring produced by an average individual during its lifetime” [26].
Mean generation time (*T*)	$T=\frac{{\ln R}_{0}}{r}$	“the time length that a population increases to *R*_0_-fold of its size at stable age-stage distribution” [26].
Age-specific survival rate (*l_x_*)	$l_{x}=\sum_{j=1}^{m}S_{xj}$	“where *m* is the number of stages” [26,37].
Age-specific fecundity (*m_x_*)	$m_{x}=\frac{\sum_{j=1}^{m}S_{xj}f_{xj}}{\sum_{j=1}^{m}S_{xj}}$	“Age-specific fecundity (*m_x_*) of the cohort at age *x*” [26,37].
Age-stage life expectancy (*e_xj_*)	$e_{xj}=\sum_{i=x}^{\infty }\sum_{y=j}^{m}{S\prime }_{iy}$	“*S*′*_iy_* is the probability that an individual of age *x* and stage *j* will survive to age *i* and stage *y* by assuming *S_xj_* = 1” [26,38].
Age-stage reproductive value (*v_xj_*)	$v_{xj}=\frac{e^{r(x+1)}}{S_{xj}}\;\sum_{i=x}^{\infty }e^{-r\left(i+1\right)}\sum_{y=j}^{m}{S\prime }_{iy}f_{iy}$	“the contribution of individuals at age *x* and stage *j* to the future population” [26,39,40].

* the population parameters table has been taken from the author’s own previous works [29,31].

**Table 2:** Toxicity of cyromazine against third-instar larvae of *Musca domestica*.

Treatment	Slope ± S.E.	LC_10_ μg/g (95% CI)	LC_25_ μg/g (95% CI)	LC_50_ μg/g (95% CI)	χ^2^ (df)	*p*
Cyromazine	1.90 ± 0.16	0.03 (0.01–0.05)	0.06 (0.04–0.09)	0.14 (0.10–0.20)	6.95 (6)	0.32

**Table 3:** The effects of lethal and sublethal concentrations of cyromazine on biological traits of *Musca domestica*.

Biological Trait	Control *	LC_10_ *	LC_25_ *	LC_50_ *
Egg hatch period (d)	1.12 ± 0.04 d	1.31 ± 0.06 c	1.48 ± 0.05 b	1.76 ± 0.06 a
Larval duration (d)	5.40 ± 0.67 d	5.96 ± 0.10 c	6.80 ± 0.13 b	7.80 ± 0.15 a
Pupal period (d)	4.33 ± 0.48 d	5.20 ± 0.10 c	6.22 ± 0.14 b	7.43 ± 0.16 a
Total preadult duration (d)	10.82 ± 0.85 d	12.44 ± 0.15 c	14.49 ± 0.22 b	17.00 ± 0.37 a
Total longevity (all individuals) (d)	36.31 ± 1.84 a	31.73 ± 1.83 b	28.66 ± 1.99 b	18.39 ± 1.95 c
Longevity (female) (d)	41.79 ± 0.66 a	39.79 ± 0.84 b	39.54 ± 0.83 b	37.12 ± 0.47 c
Longevity (male) (d)	41.30 ± 2.46 a	38.30 ± 1.57 b	38.32 ± 1.80 b	36.64 ± 0.84 c
Proportion of adult females *N_f_*/*N*	0.47 ± 0.06 a	0.40 ± 0.06 a	0.34 ± 0.06 ab	0.23 ± 0.05 b
Preoviposition period (TPOP)	17.85 ± 0.33 d	20.11 ± 0.33 c	23.12 ± 0.37 b	26.75 ± 0.41 a
Oviposition days (*O_d_*)	6.88 ± 0.39 a	6.82 ± 0.41 a	6.38 ± 0.47 a	5.56 ± 0.49 b
Fecundity (*F*) (eggs/female)	439.70 ± 17.01 a	414.00 ± 20.74 b	390.71 ± 25.91 c	332.31 ± 36.48 d
Preadult survival rate (*S_a_*)	0.85 ± 0.04 a	0.79 ± 0.07 a	0.70 ± 0.05 a	0.43 ± 0.04 b

* Values are mean ± standard error. Mean values of biological traits followed by a different alphabet in the same row are significantly different and were calculated using the paired bootstrap test.

**Table 4:** The effects of lethal and sublethal concentrations of cyromazine on population parameters of *Musca domestica*.

Parameter	Control *	LC_10_ *	LC_25_ *	LC_50_ *
Finite rate of increase (*λ*) (d^−1^)	1.27 ± 0.01 a	1.23 ± 0.01 b	1.19 ± 0.02 c	1.15 ± 0.01 d
Intrinsic rate of increase (*r*) (d^−1^)	0.24 ± 0.01 a	0.20 ± 0.02 b	0.18 ± 0.01 c	0.14 ± 0.01 d
Net reproductive rate (*R*_0_) (offspring)	207.29 ± 27.30 a	165.60 ± 25.70 b	133.96 ± 23.84 c	75.96 ± 18.50 d
Mean generation time (*T*) (d)	22.12 ± 0.47 d	24.97 ± 0.53 c	27.92 ± 0.67 b	31.00 ± 0.46 a

* Values are mean ± standard error. Mean values of population parameters followed by a different alphabet in the same row are significantly different and were calculated using the paired bootstrap test.

## Data Availability

The data presented in this study are available from the corresponding author upon reasonable request.

## References

[1] Lau K.W., Chen C.D., Lee H.L., Norma-Rashid Y., Sofian-Azirun M. (2015). Evaluation of insect growth regulators against field-collected *Aedes aegypti* and *Aedes albopictus* (Diptera: Culicidae) from Malaysia. J. Med. Entomol..

[2] Rezende-Teixeira P., Dusi R.G., Jimenez P.C., Espindola L.S., Costa-Lotufo L.V. (2022). What can we learn from commercial insecticides? Efficacy, toxicity, environmental impacts, and future developments. Environ. Pollut..

[3] Khan H.A.A., Akram W. (2017). Cyromazine resistance in a field strain of house flies, *Musca domestica* L.: Resistance risk assessment and bio-chemical mechanism. Chemosphere.

[4] Donahue Jr W.A., Showler A.T., Donahue M.W., Vinson B.E., Osbrink W.L. (2017). Lethal effects of the insect growth regulator cyromazine against three species of filth flies, *Musca domestica*, *Stomoxys calcitrans*, and *Fannia canicularis* (Diptera: Muscidae) in cattle, swine, and chicken manure. J. Econ. Entomol..

[5] Vazirianzadeh B., Jervis M., Kidd N.A. (2007). The effects of oral application of cyromazine and triflumuron on house-fly larvae. J. Arthropod-Borne Dis..

[6] Mulla M.S., Axelrod H. (1983). Evaluation of Larvadex, a new IGR for the control of pestiferous flies on poultry ranches. J. Econ. Entomol..

[7] Chen D., Zhao Y., Miao H., Wu Y. (2015). A novel dispersive micro solid phase extraction using PCX as the sorbent for the determination of melamine and cyromazine in milk and milk powder by UHPLC-HRMS/MS. Talanta.

[8] Ahmad M., Sayyed A.H., Saleem M.A., Ahmad M. (2008). Evidence for field evolved resistance to newer insecticides in *Spodoptera litura* (Lepidoptera: Noctuidae) from Pakistan. Crop. Prot..

[9] Abbas N., Shad S.A., Shah R.M. (2015). Resistance status of *Musca domestica* L. populations to neonicotinoids and insect growth regulators in Pakistan poultry facilities. Pak. J. Zool..

[10] Mansoor M.M. (2023). Risk assessment of cyromazine resistance in a field population of *Sesamia inferens* (Walker): Cross-resistance, inheritance, and realized heritability. Phytoparasitica.

[11] Khan H.A.A. (2021). Posttreatment temperature influences toxicity of insect growth regulators in *Musca domestica*. Parasitol. Res..

[12] Biondi A., Desneux N., Siscaro G., Zappalà L. (2012). Using organic-certified rather than synthetic pesticides may not be safer for biological control agents: Selectivity and side effects of 14 pesticides on the predator *Orius laevigatus*. Chemosphere.

[13] Quan L., Zhang H., Sun L., Li Y., Yan W., Yue Q., Qiu G. (2016). Research advances in sublethal effect of pesticide. J. Agric..

[14] Beers E.H., Schmidt R.A. (2014). Impacts of orchard pesticides on *Galendromus occidentalis*: Lethal and sublethal effects. Crop. Prot..

[15] Liu Z., Han Z. (2006). Fitness costs of laboratory-selected imidacloprid resistance in the brown planthopper, *Nilaparvata lugens* Stål. Pest Manag. Sci. Former. Pestic. Sci..

[16] Abbas N., Shad S.A., Razaq M. (2012). Fitness cost, cross resistance and realized heritability of resistance to imidacloprid in *Spodoptera litura* (Lepidoptera: Noctuidae). Pestic. Biochem. Physiol..

[17] Ullah F., Gul H., Desneux N., Gao X., Song D. (2019). Imidacloprid-induced hormesis effects on demographic traits of the melon aphid, *Aphis gossypii*. Entomol. Gen..

[18] Yu Y., Shen G., Zhu H., Lu Y. (2010). Imidacloprid-induced hormesis on the fecundity and juvenile hormone levels of the green peach aphid *Myzus persicae* (Sulzer). Pestic. Biochem. Physiol..

[19] Li X., Li Y., Zhu X., Li X., Cheng D., Zhang Y. (2023). Effects of imidacloprid-induced hormesis on the development and reproduction of the rose-grain aphid *Metopolophium dirhodum* (Hemiptera: Aphididae). Front. Physiol..

[20] Issa R. (2019). Musca domestica acts as transport vector hosts. Bull. Natl. Res. Cent..

[21] Kočišová A., Petrovský M., Toporčák J., Novák P. (2004). The potential of some insect growth regulators in housefly (*Musca domestica*) control. Biol. Bratisl..

[22] Khan H.A.A., Akram W., Arshad M., Hafeez F. (2016). Toxicity and resistance of field collected *Musca domestica* (Diptera: Muscidae) against insect growth regulator insecticides. Parasitol. Res..

[23] Ponnudurai G., Harikrishnan T., Rani N. (2009). In vitro evaluation of insect growth regulator cyromazine against *Musca domestica*. J Vet Parasitol.

[24] Sathiyamoorthy N., Senthilvel K., Rani N., Ramya K., Ponnudurai G. (2018). Survey on insecticide usage pattern against house fly (*Musca domestica* L.) population in commercial poultry farms in Namakkal region, Tamil Nadu, India. Studies.

[25] Jemâa J.M.-B., Boushih E. (2010). Cyromazine induced effects on larvae and adults of laboratory Tunisian strain of the Mediterranean fruit fly *Ceratitis capitata*. Tunis. J. Plant Prot..

[26] Chi H., You M., Atlıhan R., Smith C.L., Kavousi A., Özgökçe M.S., Güncan A., Tuan S.-J., Fu J.-W., Xu Y.-Y. (2020). Age-Stage, two-sex life table: An introduction to theory, data analysis, and application. Entomol. Gen..

[27] Khan H.A.A., Akram W., Shad S.A. (2013). Resistance to conventional insecticides in Pakistani populations of *Musca domestica* L.(Diptera: Muscidae): A potential ectoparasite of dairy animals. Ecotoxicology.

[28] Khan H.A.A., Shad S.A., Akram W. (2012). Effect of livestock manures on the fitness of house fly, *Musca domestica* L.(Diptera: Muscidae). Parasitol. Res..

[29] Khan H.A.A. (2021). Pyriproxyfen induces lethal and sublethal effects on biological traits and demographic growth parameters in *Musca domestica*. Ecotoxicology.

[30] Khan H.A.A. (2018). Spinosad resistance affects biological parameters of *Musca domestica* Linnaeus. Sci. Rep..

[31] Iqbal H., Fatima A., Khan H.A.A. (2022). ZnO nanoparticles produced in the culture supernatant of *Bacillus thuringiensis* ser. *israelensis* affect the demographic parameters of *Musca domestica* using the age-stage, two-sex life table. Pest Manag. Sci..

[32] Finney D. (1971). A statistical treatment of the sigmoid response curve. Probit Analysis.

[33] Chi H. (2020). Computer Program for The Age-Stage, Two-Sex Life Table Analysis.

[34] Efron B., Tibshirani R.J. (1994). An Introduction to the Bootstrap.

[35] Akca I., Ayvaz T., Yazici E., Smith C.L., Chi H. (2015). Demography and population projection of *Aphis fabae* (Hemiptera: Aphididae): With additional comments on life table research criteria. J. Econ. Entomol..

[36] Goodman D. (1982). Optimal life histories, optimal notation, and the value of reproductive value. Am. Nat..

[37] Chi H., Liu H. (1985). Two new methods for the study of insect population ecology. Bull. Inst. Zool. Acad. Sin.

[38] Chi H., Su H.-Y. (2006). Age-stage, two-sex life tables of *Aphidius gifuensis* (Ashmead) (Hymenoptera: Braconidae) and its host *Myzus persicae* (Sulzer)(Homoptera: Aphididae) with mathematical proof of the relationship between female fecundity and the net reproductive rate. Environ. Entomol..

[39] Tuan S.J., Lee C.C., Chi H. (2014). Population and damage projection of *Spodoptera litura* (F.) on peanuts (*Arachis hypogaea* L.) under different conditions using the age—Stage, two—Sex life table. Pest Manag. Sci..

[40] Fisher R., Bennett J.H. (1930). The Genetical Theory of Natural Selection: A Complete Variorum Edition.

[41] Shapiro–Ilan D.I., Cottrell T.E., Jackson M.A., Wood B.W. (2013). Control of key pecan insect pests using biorational pesticides. J. Econ. Entomol..

[42] Khater H.F. (2012). Ecosmart biorational insecticides: Alternative insect control strategies. Insectic. Adv. Integr. Pest Manag..

[43] Francesena N., Schneider M.I. (2018). Selectivity assessment of two biorational insecticides, azadirachtin and pyriproxyfen, in comparison to a neonicotinoid, acetamiprid, on pupae and adults of a Neotropical strain Eretmocerus mundus Mercet. Chemosphere.

[44] Haddi K., Turchen L.M., Viteri Jumbo L.O., Guedes R.N., Pereira E.J., Aguiar R.W., Oliveira E.E. (2020). Rethinking biorational insecticides for pest management: Unintended effects and consequences. Pest Manag. Sci..

[45] Ghoneim K., Tanani M., Hamadah K., Basiouny A., Waheeb H. (2015). Effects of Novaluron and Cyromazine, chitin synthesis inhibitors, on the larval haemogram of *Spodoptera littoralis* (Boisd.) (Lepidoptera: Noctuidae). Int. J. Adv. Res..

[46] Tanani M., Ghoneim K., Hamadah K., Basiouny A., Waheeb H. (2016). Disruptive effects of some novel chitin synthesis inhibitors on the transaminase activity in larval tissues of *Spodoptera littoralis*(Lepidoptera: Noctuidae). Int. J. Res. Stud. Zool..

[47] Taylor D.B., Friesen K., Zhu J.J., Sievert K. (2012). Efficacy of cyromazine to control immature stable flies (Diptera: Muscidae) developing in winter hay feeding sites. J. Econ. Entomol..

[48] Shah R.M., Alam M., Ahmad D., Waqas M., Ali Q., Binyamin M., Shad S.A. (2016). Toxicity of 25 synthetic insecticides to the field population of *Culex quinquefasciatus* Say. Parasitol. Res..

[49] Awad T.I., Mulla M.S. (1984). Morphogenetic and histopathological effects of the insect growth regulator cyromazine in larvae of *Culex quinquefasciatus* (Diptera: Culicidae). J. Med. Entomol..

[50] Elia-Amira N., Chen C., Low V., Lau K., Haziqah-Rashid A., Amelia-Yap Z., Lee H., Sofian-Azirun M. (2022). Statewide efficacy assessment of insect growth regulators against *Aedes albopictus* (diptera: Culicidae) in Sabah, Malaysia: An alternative control strategy?. J. Med. Entomol..

[51] Lau K.W., Chen C.D., Lee H.L., Low V.L., Sofian-Azirun M. (2018). Bioefficacy of insect growth regulators against *Aedes albopictus* (Diptera: Culicidea) from Sarawak, Malaysia: A statewide survey. J. Econ. Entomol..

[52] Van De Wouw A.P., Batterham P., Daborn P.J. (2006). The insect growth regulator insecticide cyromazine causes earlier emergence in *Drosophila melanogaster*. Arch. Insect Biochem. Physiol..

[53] Weintraub P.G. (2001). Effects of cyromazine and abamectin on the pea leafminer *Liriomyza huidobrensis* (Diptera: Agromyzidae) and its parasitoid *Diglyphus isaea* (Hymenoptera: Eulophidae) in potatoes. Crop. Prot..

[54] Fontes J., Roja I.S., Tavares J., Oliveira L. (2018). Lethal and sublethal effects of various pesticides on *Trichogramma achaeae* (Hymenoptera: Trichogrammatidae). J. Econ. Entomol..

[55] Cetin H., Erler F., Yanikoglu A. (2009). Survey of insect growth regulator (IGR) resistance in house flies (*Musca domestica* L.) from southwestern Turkey. J. Vector Ecol..

[56] Desneux N., Decourtye A., Delpuech J.-M. (2007). The sublethal effects of pesticides on beneficial arthropods. Annu. Rev. Entomol..

[57] Müller T., Prosche A., Müller C. (2017). Sublethal insecticide exposure affects reproduction, chemical phenotype as well as offspring development and antennae symmetry of a leaf beetle. Environ. Pollut..

[58] Hannig G.T., Ziegler M., Marcon P.G. (2009). Feeding cessation effects of chlorantraniliprole, a new anthranilic diamide insecticide, in comparison with several insecticides in distinct chemical classes and mode-of-action groups. Pest Manag. Sci. Former. Pestic. Sci..

[59] Shen N., Liu H.Y., Mou T.Y., Ma Y.B., Li Y., Song Z.J., Tang T., Han Z.J., Zhao C.Q. (2022). Novel meta-diamide insecticide, broflanilide, suppresses the population of common cutworm *Spodoptera litura* through its lethal and sublethal effects. Pest Manag. Sci..

[60] Gope A., Chakraborty G., Ghosh S.M., Sau S., Mondal K., Biswas A., Sarkar S., Sarkar P.K., Roy D. (2022). Toxicity and sublethal effects of fluxametamide on the key biological parameters and life history traits of diamondback moth *Plutella xylostella* (L.). Agronomy.

[61] Zhang S.-W., Zeng Q.-H., Yang H., Zhang C., Ding B., Yang H.-Z., Yang M.-F. (2023). Sublethal and transgenerational effects of broflanilide on *Myzus persicae* (Sulzer) (Hemiptera: Aphididae). Crop. Prot..

[62] Yasoob H., Ali Khan H.A., Zhang Y. (2017). Toxicity and sublethal effects of cantharidin on *Musca domestica* (Diptera: Muscidae). J. Econ. Entomol..

[63] Wang S., Qi Y., Desneux N., Shi X., Biondi A., Gao X. (2017). Sublethal and transgenerational effects of short-term and chronic exposures to the neonicotinoid nitenpyram on the cotton aphid *Aphis gossypii*. J. Pest Sci..

[64] Guedes R.N.C., Rix R.R., Cutler G.C. (2022). Pesticide-induced hormesis in arthropods: Towards biological systems. Curr. Opin. Toxicol..

[65] Deng D., Duan W., Wang H., Zhang K., Guo J., Yuan L., Wang L., Wu S. (2019). Assessment of the effects of lethal and sublethal exposure to dinotefuran on the wheat aphid *Rhopalosiphum padi* (Linnaeus). Ecotoxicology.

[66] Zhou C., Yang X.-b., Yang H., Long G.-y., Jin D.-c. (2020). Effects of sublethal concentrations of insecticides on the fecundity of *Sogatella furcifera* (Hemiptera: Delphacidae) via the regulation of vitellogenin and its receptor. J. Insect Sci..

[67] Budia F., Viñuela E. (1996). Effects of cyromazine on adult *C. capitata* (Diptera: Tephritidae) on mortality and reproduction. J. Econ. Entomol..

[68] Pinto M.C., Prado A.P.d. (2001). Resistance of *Musca domestica* L. populations to cyromazine (insect growth regulator) in Brazil. Mem. Inst. Oswaldo Cruz.

[69] Acevedo G.R., Zapater M., Toloza A.C. (2009). Insecticide resistance of house fly, *Musca domestica* (L.) from Argentina. Parasitol. Res..

[70] Bell H.A., Robinson K.A., Weaver R.J. (2010). First report of cyromazine resistance in a population of UK house fly (*Musca domestica*) associated with intensive livestock production. Pest Manag. Sci..

[71] Chi H. (1988). Life-table analysis incorporating both sexes and variable development rates among individuals. Environ. Entomol..

[72] Akköprü E.P., Atlıhan R., Okut H., Chi H. (2015). Demographic assessment of plant cultivar resistance to insect pests: A case study of the dusky-veined walnut aphid (Hemiptera: Callaphididae) on five walnut cultivars. J. Econ. Entomol..

